# A Heart Stopping Case of the Bezold-Jarisch Reflex

**DOI:** 10.1155/2015/359401

**Published:** 2015-12-01

**Authors:** Marc-Etienne Parent, Serge Lepage

**Affiliations:** ^1^Department of Internal Medicine, Centre Hospitalier Universitaire de Sherbrooke, 3001 12^e^ Avenue, Fleurimont, QC, Canada J1H 5N4; ^2^Department of Cardiology, Centre Hospitalier Universitaire de Sherbrooke, 3001 12^e^ Avenue, Fleurimont, QC, Canada J1H 5N4

## Abstract

The Bezold-Jarisch reflex is a parasympathetic reflex induced by intense mechanical stimulation of the ventricular myocytes. Exceptionally, cases have been described in patients receiving dobutamine infusion during a stress echocardiography. All were healthy middle-aged women and recovered without sequelae. A healthy 60-year-old woman suffered two 5.9-second episodes of asystole during her 20 mcg/kg/min infusion of dobutamine. Recovery was quick and without sequelae. Echocardiography and coronary angiography were both normal. In conclusion, this is the fourth documented case of a severe Bezold-Jarisch reflex causing asystole during dobutamine infusion. Diagnosis can only be made after excluding all other possible diagnoses, most importantly ischemia. This serves as a reminder of the importance of close monitoring during dobutamine infusion.

## 1. Introduction

The Bezold-Jarisch reflex is an inhibitory reflex induced by the stimulation of mechanoreceptors in the heart, be it chemical or mechanical. It promotes parasympathetic activity, leading to bradycardia, vasodilation, and hypotension. This reflex is thought to be the basis of some unusual reactions where hyper stimulation of the myocardium paradoxically leads to profound bradycardia or asystole.

Asystole is an excessively rare complication of dobutamine echocardiography testing, with a documented incidence of 3 cases in over 83 000 patients. Patients are twice as likely to suffer from cerebrovascular accidents, cardiac rupture, or even death than from asystole according to the numbers in a 2010 review [1].

## 2. Case Report

A 60-year-old Caucasian female was referred to the cardiology department for a dobutamine stress echocardiography as part of the investigation for activity related chest pain. Past medical history revealed hypertension, dyslipidemia, and gastroesophageal reflux treated with valsartan 80 mg daily, rosuvastatin 20 mg daily, and pantoloc 40 mg daily, respectively. Family history was positive for cardiovascular disease. In the prior month, an exercise stress test was inconclusive, reaching only 69% of her predicted maximal heart rate. The test was halted for fatigue and dizziness. Blood pressure was normal both during and after the examination. No chest pain was present. Despite the suboptimal effort, a 1 mm ST depression was noted in the inferior lateral leads.

**Figure 1:**
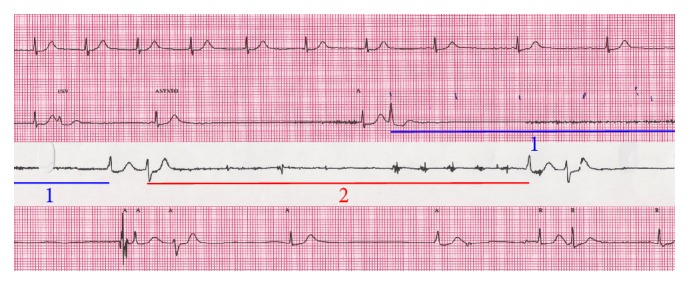
Continuous electrocardiographic recording of lead 1 at a speed of 25 mm/s showing progressive bradycardia leading to two episodes (1 and 2) of asystole both lasting 5.9 seconds, followed by a progressive return to sinus rhythm. On the second line, the first wave shows a premature atrial contraction while the third wave shows a premature junctional complex. On the third line, cardiac massage is noted. On the fourth line, the fourth wave is showing fusion of the P and QRS waves.

During the echocardiography, dobutamine was infused in three sequences: 5, 10, and 20 mcg/kg/min. Echography performed during the infusion showed a progressive and diffuse increase in heart contractility. Shortly after reaching the 20 mcg/kg/min infusion rate, the patient developed sinus bradycardia as well as sinus arrhythmia, rapidly followed by asystole (Figure 1). Dobutamine infusion was ceased. Spontaneous normal sinus rhythm resumed following a short sequence of chest compressions and administration of intravenous atropine 1 mg. The patient rapidly developed sinus tachycardia, at a rate of 150 beats per minute, before returning to normal sinus rhythm (not shown in Figure 1). Recovery was without sequelae. Immediately after the event, echocardiography showed normal cardiac anatomy and segmental contractility, an absence of valvulopathy, and a left ventricular ejection fraction of 70%. The following day, coronary angiography was completely normal. The patient's heart rhythm was monitored for 48 hours and showed normal sinus rhythm without tachy- or bradyarrhythmia. With a follow-up of over 6 months, the patient remains well and asymptomatic.

## 3. Discussion

The three documented cases, all of which survived without sequelae, were described in case reports and are discussed briefly here. The first case was a 60-year-old woman with baseline T wave changes on her EKG. During a 20 mcg/kg/min dobutamine infusion, she developed hypertension followed by bradycardia and asystole of more than 8 seconds. Diffuse hyperkinesia was evident prior to the asystole. Coronary angiography was not performed due to patient refusal [2]. The second case was a healthy 48-year-old woman with equivocal findings during bicycle ergometry. During the 40 mcg/kg/h dobutamine infusion, she developed idioventricular rhythm followed by “prolonged” asystole. Sinus rhythm promptly returned following cardiac massage. Echocardiography, coronary angiography, and 7 days of cardiac monitoring were normal [3]. The third case was a 59-year-old woman with a positive treadmill test for ischemia. Soon after reaching an infusion rate of 30 mcg/kg/min she developed nonsustained ventricular tachycardia, bradycardia, and, finally, asystole lasting 8.4 seconds. The echocardiography, done simultaneously, was normal, as was coronary angiography on the next day [4].

In a 1998 review on the safety of dobutamine echocardiography in 3041 patients, Pezzano et al. [5] reported a single transient asymptomatic sinus pause lasting 6 seconds. No further information is available concerning this case.

A review of literature covering the year 2010 and beyond did not uncover any additional cases. The Bezold-Jarisch reflex is often incriminated as a cause of bradycardia or hypotension in various clinical settings such as stress testing [6] and neuraxial anesthesia. However, its role in cases of neuraxial anesthesia induced cardiac arrest remains uncertain [7, 8].

Before the Bezold-Jarisch reflex can be considered as the cause of asystole, other diagnoses need to be eliminated. Inferior ischemia causing sinoatrial or atrioventricular nodal dysfunctions must be ruled out. Diagnoses such as arrhythmia, valvulopathies, and other causes of ventricular outflow tract obstruction must also be sought out. The normal angiography rules out static obstructions as caused by atherosclerosis or thrombi. Ischemia caused by a dynamic obstruction, such as a coronary vasospasm, would have been associated with abnormal segmental contractility on echocardiography. The progressive increase in contractility shows adequate response to dobutamine. The normal cardiac monitoring and atropine-induced sinus tachycardia both demonstrate adequate nodal function in this patient. The fact that bradycardia with sinus arrhythmia occurred prior to the event, during maximal dobutamine infusion and contractility, also supports the vagal nature of this phenomenon.

The development of a severe Bezold-Jarisch reflex leading to asystole during dobutamine stimulation is extremely rare and seemingly benign. This is most likely a self-resolving phenomenon and requires no specific treatment, other than dobutamine cessation. However, when faced with cardiac arrest, all patients should be promptly treated and investigated to rule out potentially fatal underlying causes. This is, to our knowledge, the fourth reported case of asystole during a dobutamine stress echocardiography. Interestingly, all documented cases were previously healthy women with relatively similar ages and normal—for those who did undergo the procedure—coronary angiographies. It is important, when considering various stress testing modalities, that benefits be carefully weighed against possible risks. When possible, exercise stress testing should be favored as it allows clinicians to perform a simultaneous assessment of the patient's effort tolerance. These severe but rare cases remind us of the potential complications and importance of closely monitoring patients during dobutamine infusion.
